# Metastases to the pituitary gland: insights from the German pituitary tumor registry

**DOI:** 10.1007/s11102-023-01361-0

**Published:** 2023-10-30

**Authors:** Linus Haberbosch, Simone Schmid, Vanessa Hubertus, Dominik Soll, Güliz Acker, Matthias Dottermusch, Marie Jensen, Lukas Maurer, Joachim Spranger, Knut Mai, Peter Vajkoczy, Wolfgang Saeger, Christian J. Strasburger

**Affiliations:** 1 Department of Endocrinology and Metabolism, Charité—Universitätsmedizin Berlin, corporate member of Freie Universität Berlin, Humboldt-Universität zu Berlin, Berlin, 10117 Germany; 2 Pituitary Tumor Center of Excellence, Charité—Universitätsmedizin Berlin, corporate member of Freie Universität Berlin, Humboldt-Universität zu Berlin, Berlin Institute of Health, Charitéplatz 1, Berlin, 10117 Germany; 3 BIH Charité Junior Digital Clinician Scientist Program, Berlin Institute of Health at Charité—Universitätsmedizin Berlin, BIH Biomedical Innovation Academy, Charitéplatz 1, Berlin, 10117 Germany; 4 Department of Neuropathology, Charité—Universitätsmedizin Berlin, corporate member of Freie Universität Berlin, Humboldt-Universität zu Berlin, Berlin, 10117 Germany; 5 Department of Neurosurgery, Charité—Universitätsmedizin Berlin, corporate member of Freie Universität Berlin, Humboldt-Universität zu Berlin, Berlin, 10117 Germany; 6 BIH Charité Clinician Scientist Program, Berlin Institute of Health at Charité—Universitätsmedizin Berlin, BIH Biomedical Innovation Academy, Charitéplatz 1, Berlin, 10117 Germany; 7 Department of Radiation Oncology and Radiotherapy, Charité—Universitätsmedizin Berlin, corporate member of Freie Universität Berlin, Humboldt-Universität zu Berlin, Berlin, 10117 Germany; 8 Institute of Neuropathology, University Medical Center, Hamburg-Eppendorf, Hamburg, Germany; 9 Institute of Pathology, University Medical Center, Hamburg-Eppendorf, Hamburg, Germany

## Abstract

Metastatic involvement of the pituitary gland is a rare but clinically significant phenomenon, that often poses diagnostic and therapeutic challenges. The aim of this study was to provide a comprehensive analysis of the origin of pituitary metastases using data from the German Pituitary Tumor Registry, one of the globally largest collections of pituitary pathology specimens. Here, we report data from a retrospective analysis of patients with metastases to the pituitary registered between 1990 and 2022. Out of 17,896 pituitary cases in the registry during this period, a total of 96 metastases to the pituitary gland were identified, accounting for 0.5% of all pituitary tumors in the registry. The mean age of the patients was 64 years. Breast cancer was identified as the primary tumor in 25% of total cases (n = 24/96) and in 50% of female patients. The second most prevalent primary tumor was lung cancer (18.75%, n = 18/96), followed by renal cell carcinoma (14.58%, n = 14/96). In comparison to current meta-analyses, this cohort shows a higher prevalence of metastases originating from the kidney. Furthermore, in contrast to the existing literature, no case of primary thyroid tumor was identified. Our study highlights the importance of pituitary metastases as a differential diagnosis in patients presenting with pituitary tumors.

**Supplementary Information:**

The online version contains supplementary material available at 10.1007/s11102-023-01361-0.

## Introduction

The pituitary gland is a known, but uncommon, site for metastatic spread of systemic solid organ and hematopoietic neoplasms. According to previous analyses, pituitary metastases account for an estimated 1% of all pituitary tumor surgical resections, but less than 1% of all cranial metastases [1]. Due to endocrine complications that impact survival and quality of life in already severely affected patients with metastatic cancer, early detection and appropriate treatment of these metastases is critically important [2]. However, due to the relative rarity of this entity, there are few larger studies of pituitary metastases, and its clinical and radiological diagnosis is often based on an already identified primary tumor that is also likely to metastasize to the pituitary.

Tumors can spread to the pituitary gland either through the abundant blood supply or by extending from nearby areas in the skull base [3, 4]. They can also spread from the space around the pituitary gland via the meningeal system.

The posterior lobe of the pituitary is more frequently affected than the anterior lobe [4, 5]. This may be due to the more direct link of the posterior lobe to the systemic circulation, as opposed to the anterior lobe, which obtains arterial blood supply predominantly through the hypophyseal portal system [3, 6]. Due to its smaller size in comparison to the anterior pituitary, the presence of the same amount of metastatic tissue in the posterior region leads to earlier clinical symptoms [7, 8], manifesting with arginine-vasopressin (AVP) deficiency [9], which is otherwise relatively rare in pituitary neuroendocrine tumors (PitNETs)/adenomas.

Pathological identification of the neoplasm may prove difficult, particularly in the case of neuroendocrine tumors, which could be mistaken for null cell PitNET/adenomas [10]. Identification of the primary tumor often requires the combination of clinical appearance and pathological diagnostics. In recent meta-analyses, breast and lung carcinomas were responsible for most of the metastases to the sellar region [7, 9]. However, because most of the studies included in these meta-analyses are small case series, larger datasets are needed to improve our understanding of pituitary metastases.

The German Pituitary Tumor registry is a large database of pituitary pathology findings from the German reference center in Hamburg, containing 17,896 pituitary pathology cases from our investigation period of 1990–2022. In an earlier audit of the same registry in 2007, 23 cases of metastases to the pituitary were identified [1], and a recent publication from the same database on multiple malignant lesions reported twelve cases of combined PitNET/adenoma and metastasis in the pituitary gland [11].

Given the lack of extensive recent studies on pituitary metastases, our aim was to extract the histopathological reports from this major registry and report the frequency of primary tumors. We also investigated the sub cohort of autopsy findings with respect to tissue invasion.

## Methods

For our study, the cases were extracted from the files of the German Pituitary Tumor Registry of the German Society for Endocrinology from 1990 up to 2022. The data was analyzed and discussed in close collaboration between neuropathologists, endocrinologists, neurosurgeons and radiologists as recommended by the Pituitary tumor center of excellence (PTCOE) initiative [12, 13]. Analyses of patient characteristics, primary tumors and tissue invasion were performed in a descriptive manner according to data availability.

## Results

### Cases

Via manual search within the German Pituitary Tumor Registry database, 96 cases of metastasis to the pituitary were identified, of which 73 were surgical cases (76%) and 23 were autopsy cases (24%).

### Primary malignancy

**Fig. 1:**
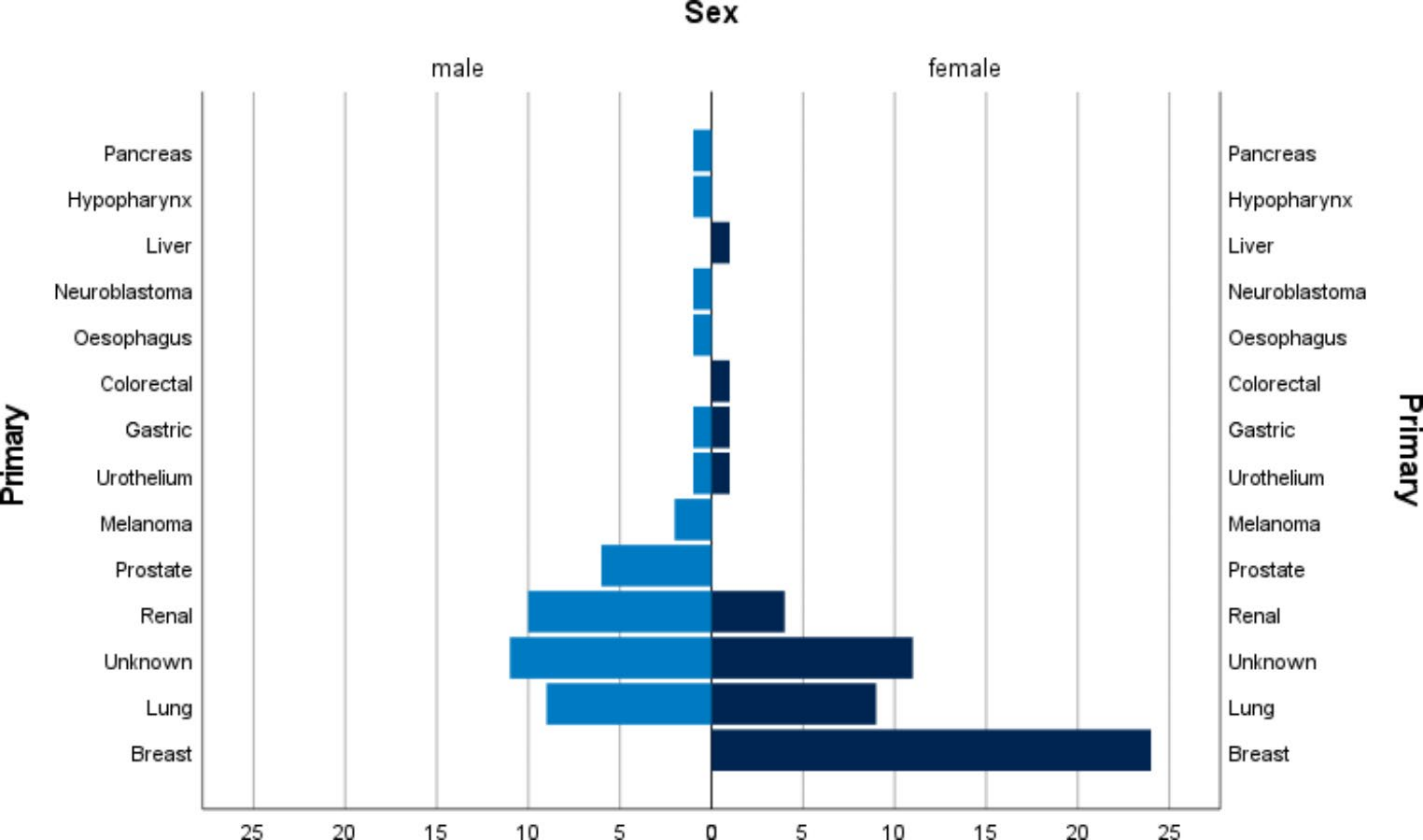
Primary tumors of metastases to the sellar region—Overview by sex. All metastases to the sellar region from the German Pituitary Tumor registry listed by primary tumor. The x-axis depicts the number of patients. “Unknown” is defined as no clear specification of a primary tumor. A list of all metastases with unknown primary tumors is provided in Supplementary Table 1

Breast cancer was identified as the primary tumor in 25% of all cases (n = 24/96) and was the origin in 50% of affected female patients. Lung cancer was the second most prevalent primary tumor (n = 18/96, 18.75%), followed by renal cell carcinoma (n = 14/96, 14.58%). Prostate cancer was the fourth most abundant tumor entity overall and third most frequent in male patients. We identified two cases each of primary tumors originating from the stomach (one adenocarcinoma, one scirrhous carcinoma), the urothelium and the skin (melanoma). Singular cases of liver cancer (hepatocellular carcinoma), pancreatic cancer (adenocarcinoma), hypopharyngeal (squamous cell carcinoma), esophageal (poorly differentiated, solid carcinoma), colorectal cancer (neuroendocrine carcinoma) and neuroblastoma were identified. The results are shown in Table 1. Figure 1 presents the frequency of tumor entities divided by sex.

**Table 1:** Primary tumors of metastases to the sellar region

Primary	Cases	Percent
Breast	24	25.0%
Unknown*	22	22.9%
Lung	18	18.8%
Renal	14	14.6%
Prostate	6	6.3%
Gastric	2	2.1%
Urothelium	2	2.1%
Melanoma	2	2.1%
Liver	1	1.0%
Pancreas	1	1.0%
Hypopharynx	1	1.0%
Oesophagus	1	1.0%
Rectal	1	1.0%
Neuroblastoma	1	1.0%
Total	96	100%

All metastases to the sellar region from the German Pituitary Tumor registry listed by primary tumor. * Unknown defined as no clear specification of a primary tumor. A list of all metastases with unknown primaries is provided in Supplementary Table 1

In 22 exclusively neurosurgical cases, the primary tumor remained unidentified. This was mostly due to the difficulty in differentiation between on the one hand primary cancer of the pituitary or metastases of poorly differentiated tumors and between metastasis and infiltration (e.g. squamous cell carcinoma) on the other hand not being possible due to lack of further clinical data or tissue. A list of these cases is provided in Supplementary Table 1.

Attempting to differentiate between diagnoses derived from autopsy on the one hand and neurosurgical cases on the other, we found that unknown origin was only described in the neurosurgical cases, whereas gastric, esophageal and pancreatic cancer origins were only described in autopsy cases. Interestingly, renal cell carcinoma was only described in neurosurgical cases.

### Demographics

**Fig. 2:**
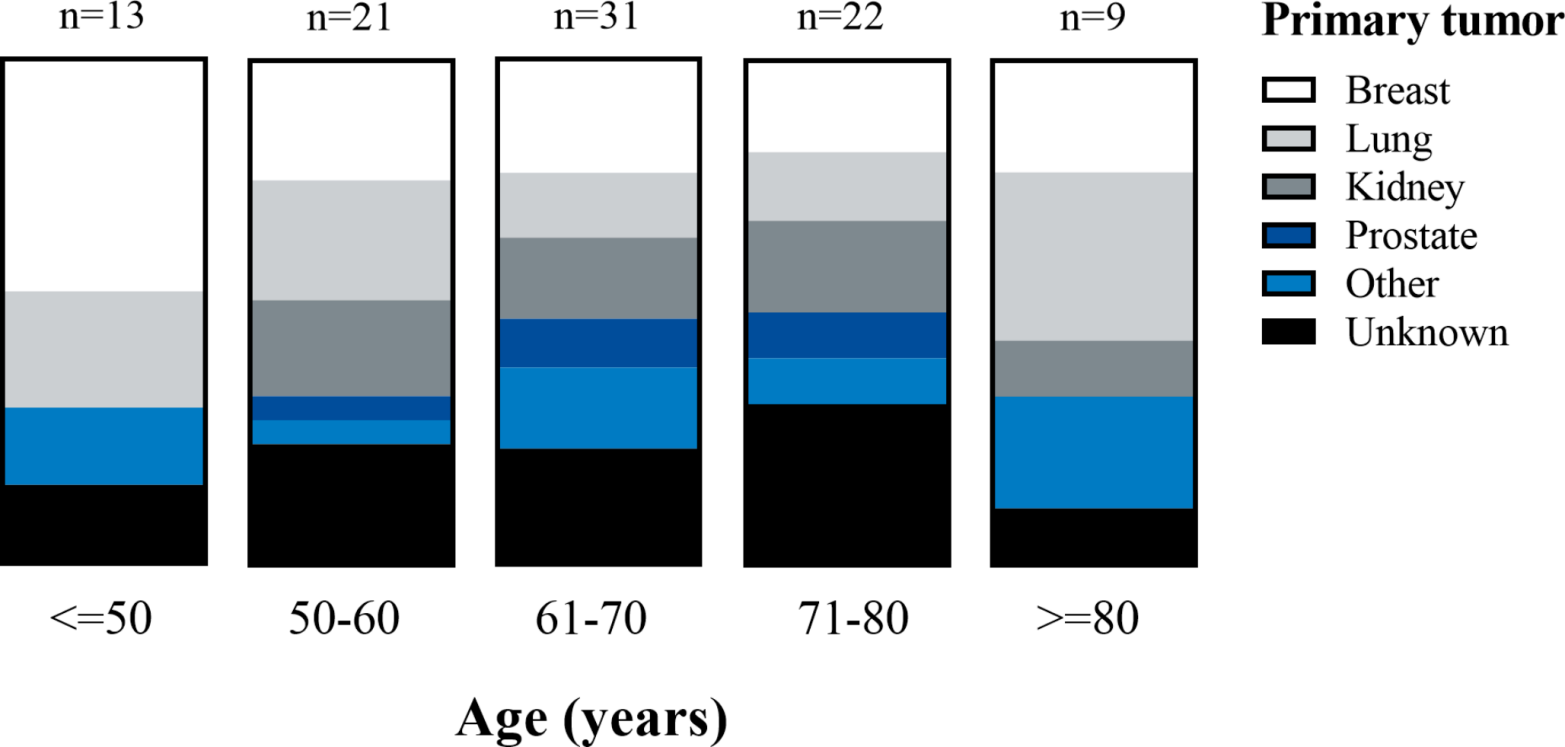
Primary tumors of metastases to the sella region—Overview by age. Relative distribution of primary tumors of metastases to the sellar region from the German Pituitary Tumor registry divided by age group. The x-axis depicts age intervals, with the number of subjects in each interval category listed above the corresponding bar. The category “Unknown” is defined as no clear specification of a primary tumor. A list of all metastases with unknown primaries is provided in Supplementary Table 1

The patients affected by metastases to the pituitary were almost equally female and male, with a median age of 64 years. The youngest patient was 11 years old (primary neuroblastoma), the oldest 92 (primary urothelial carcinoma). A breakdown of the primary tumors by age is presented in Fig. 2.

### Infiltration

**Fig. 3:**
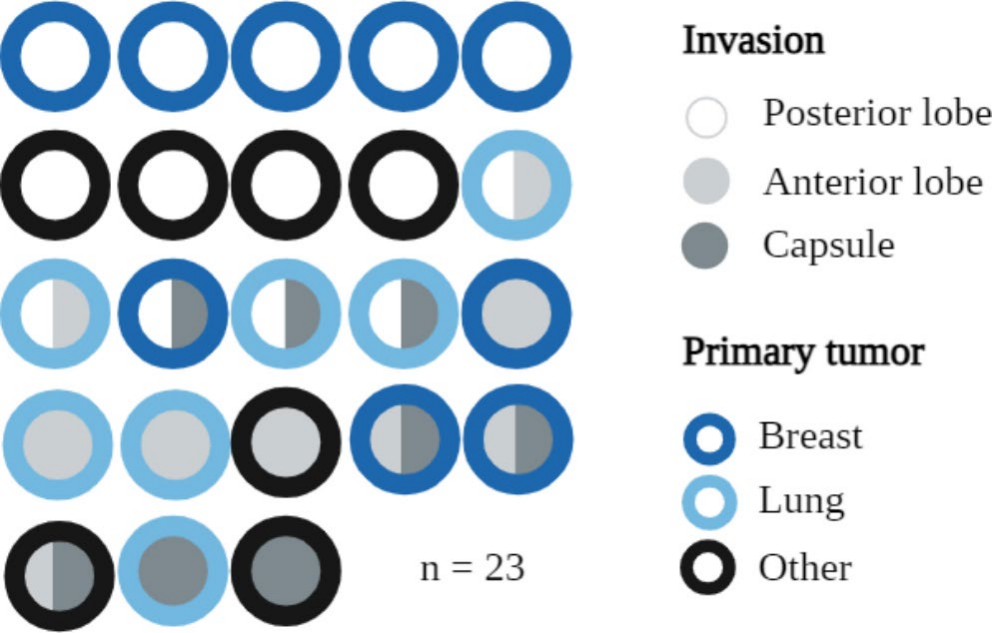
Tissue invasions in the autopsy cohort. All tumors from the autopsy cohort from the German Pituitary Tumor registry listed by tissue invasion. The fill color discerns tissue invasion, the rim color the primary tumor

In the autopsy sub-cohort (n = 23), we detected 9 cases of posterior lobe invasion, 2 cases of combined anterior and posterior lobe invasion, 3 cases of combined posterior lobe and capsule invasion, 4 cases of anterior lobe invasion, 3 cases of combined anterior lobe and capsule invasion and finally 2 cases of capsule invasion only. The findings are presented in Fig. 3.

## Discussion

In this comprehensive analysis of the German Pituitary Tumor Registry, we show a prevalence and primary distribution of metastases to the pituitary largely consistent with previously published literature of mostly smaller case series [14]. The identified primary tumor entities clearly point towards a hematogenic rather than lymphogenic mode of metastasis formation for the metastases found in the sellar region. Furthermore, primary tumors known to metastasize to the bone represent a majority of the pituitary lesions identified as metastases.

In comparison to recent meta-analyses [7, 9], our cohort is lacking representation of thyroid primary tumors but shows a higher rate of renal cell carcinoma primary tumors.

This study is limited due to the scarcity of available clinical data. Furthermore, an extensive re-evaluation of historical cases with more recent histopathological methods was not performed and was not the intent of this analysis. However, the size of the case database analyzed here holds significant importance. Given the lack of extensive current studies on metastases to the pituitary gland, our findings highlight the need for further large cohort studies that integrate clinical, pathological and radiological findings.

### Prevalence of metastases

The 96 metastases represent a total of 0.5% of the cases included in the German Pituitary Tumor Registry (N = 17,896), which is consistent with a previous report from 2007 to 25 cases (0.6%) [1]. This confirms the rarity of metastases and underscores the importance of registries to record such cases.

### Primary cancer location

The finding of breast and lung cancer as the two most prevalent primary tumors in our cohort confirms the most recent meta-analyses [8, 9, 15].

Breast cancer is with 25% of cases the most common primary in our study as well as the literature, representing 24–26% of cases in the meta-analyses of Ng et al. and Javanbakht et al. [7, 9]. This is likely due to the propensity of breast cancer to metastasize to the bone as well as possibly the prolactin-rich environment of the pituitary [7]. While systemic prolactin does not seem to facilitate breast cancer growth, paracrine (as well as autocrine) prolactin stimulation is an important factor in mammary tumor formation [16].

The second most common primary tumor in our study was lung cancer. Considering its general prevalence and predisposition to metastasize to bone and brain [17, 18], lung cancer is the most or second most common primary tumor in pituitary metastases meta-analyses and the most common primary tumor in men [7, 9, 19].

Remarkably, colorectal cancer, which also represents one of the more common malignancies along with lung and breast cancer, rarely presented as pituitary metastases in our study. This is in line with previous reports [7] and underlines that the mechanism of metastatic spread to the pituitary region mostly originates from the vascular system surrounding the primary tumor.

Pituitary metastases have been identified as the initial manifestation of a previously unknown malignant tumor in 20–40% of tumors neurosurgically removed from the sellar region [19–22], which is well in keeping with our data showing an unknown primary tumor in 22.9% of cases. One of the key challenges of pathological analyses of surgical tissue is the integration of clinical and histopathological findings. If the patient has no previously known neoplastic disease, or one of an entity which is not prone to osseous metastasis, a definitive characterization of the tissue as metastatic is often not possible. It should be noted that due to the historical nature of the registry, a number of cases classified as unknown primary tumor, today might be identified with more modern histopathological diagnostic methods.

Notably, our case series of metastases to the pituitary gland contained a higher number of renal cell carcinomas than most previously described case series, but no case of thyroid carcinoma. This is surprising, as both cancers tend to metastasize to the bone and show similar rates of growth [23, 24]. The suggested work-up and staging guidelines for both renal cell and thyroid carcinoma also do not contain a routine cranial MRI, despite this being proposed for metastatic renal cell carcinoma [25]. Regarding renal cell carcinoma, most of the cases have been diagnosed in the years 2009–2022, possibly representing a higher histological diagnostic certainty based on novel antigen markers. These hypotheses, however, remain speculative, as a comprehensive comparison of available antigen markers and re-analysis of tumor tissue from older samples was not possible.

A recent review did not mention thyroid metastases to the pituitary [8], and it has historically been reported as very rare [26]. However, a meta-analysis from the same year reported a comparatively high rate of thyroid primary tumors in 4.5% [9]. Thyroid cancer might be diagnosed earlier because of the abundant availability of thyroid ultrasound and the comparatively aggressive diagnostic and therapeutic approach in Germany as an endemic iodine deficiency area with a high prevalence of regressive thyroid nodules. This higher surveillance rate in turn may lead to a lower rate of metastatic thyroid cancer compared to other countries [27]. In addition to the possible earlier diagnosis and resection of thyroid cancer, according to German recommendations, a radioiodine therapy is performed in earlier thyroid cancer stages compared to e.g. American guidelines, possibly leading to a lower incidence of pituitary metastases from thyroid cancer [28–30].

The data presented in this analysis should alert clinicians to consider the possibility of metastasis in patients with any of the listed neoplastic entities and a newly detected sellar mass, particularly if the primary tumor has already metastasized to the bone [31].

### Invasion

In an analysis of the autopsy subgroup, we confirmed the current perspective on the higher prevalence of invasion to the posterior as opposed to the anterior pituitary. There are different hypotheses explaining this phenomenon. The posterior gland is directly connected to systemic circulation, while the hypophyseal portal system may be protecting the anterior gland to a certain degree. A pituitary metastasis might also be secondary to a sellar bone metastasis, which spreads to the pituitary gland tissue via existing vascular contacts. The latter view is supported by an autopsy study showing pituitary and sellar bone metastases, rather than brain metastases, to be syntopic [31]. It is further corroborated by a recent study discussing combinations of pituitary tumors and metastases [11].

### Clinical considerations

As sellar masses are often discovered incidentally, clinical criteria to assess the likelihood of metastasis are required to guide (radio-)surgical decision-making. While PitNET/adenomas are common in the general population, with a prevalence of microadenoma of about 10% in thon MRI imaging, it should be noted that in patients with a known malignant tumor, a pituitary mass is more likely to represent a metastasis (incidence reported around 5%) than a PitNET/adenoma (incidence of 1.8%) [3–5, 20]. This information should also be provided to the neuropathologists for consideration in their assessment. As metastases are more likely to affect the posterior pituitary, the defining clinical symptom differentiating pituitary metastasis from PitNET/adenoma is isolated AVP deficiency [7].

In addition to clinical presentation, imaging diagnostics can help to identify pituitary metastases. However, signal characteristics of metastases on MRI are variable. Findings include loss of hyperintensity of the posterior pituitary (pituitary bright spot) on T1 weighted imaging [32, 33] and hyperintensity on T2 [3], although reports are conflicting [32, 33]. Postgadolinium enhancement is also not reported consistently [32]. The European Society of Endocrinology guidelines for the management of aggressive pituitary tumors suggest an initial imaging protocol comprising of 2–3 mm sagittal T1, coronal T1 before and after gadolinium injection, coronal T2 or axial T1-weighted slices to assess a possibly malignant mass in the sellar region [34].

Rapid growth on follow-up MRI can be a warning sign for a metastasis, sometimes with a constriction by the sellar diaphragm leading to a dumbbell-shaped tumor [32, 35]. While cavernous sinus invasion is also common in PitNET/adenomas, a bilateral invasion can point towards malignancy [33]. Furthermore, the presence of visual pathway edema enhances the likelihood of a metastasis over a pituitary macroadenoma [36].

While bony erosion of the sellar floor can also occur in PitNET/adenomas, it may occur more frequently in metastasis [33]. Haemorrhage and apoplexy are uncommon [33]. There are currently no conclusive reports on the utility of molecular imaging in pituitary metastases.

While neurosurgery is an important treatment option, gross total resection of the tumor is often impossible due to infiltration of the surrounding tissue and bone as well as intense vascularization [37]. Although survival rates in cancer patients with pituitary metastasis are known to be poor, clinical case series have shown prolonged survival in patients with certain pathologies due to surgical removal of the pituitary metastasis [36, 38, 39]. However, as the therapy goal in those patients often is of palliative nature, and additionally, when there is no acute cranial nerve compression, radiosurgery can be an effective alternative to neurosurgery in selected cases [40–42], and has become the first-line treatment in many centers [36, 43].Importantly, a recent retrospective study by Hong et al. showed reduced overall survival in patients with non-treated pituitary metastases [44]. Their study reported on 48 cases, which is a large cohort considering the rarity of these entities. Still, the limited number of cases, the retrospective nature of this study and possible selection bias do not allow for a clear conclusion on treatment recommendation.

In our center, we generally recommend a combined hybrid procedure of surgery and radiosurgery or conventional radiotherapy in the treatment of metastases to the pituitary, since the required radiation dose differs between PitNET/adenomas and a metastases, and imaging alone is not sufficient to obliviate pathologic evaluation.

## Conclusion

In conclusion, our analysis from a large registry provides a suitable basis for an assessment of the incidence of metastatic disease in Germany and illuminates the differential diagnosis in pituitary tumors with important information on the distribution among different entities. Further multicenter studies are needed to inform treatment decisions.

### Electronic supplementary material

Below is the link to the electronic supplementary material.

Supplementary Material 1

## Data Availability

The anonymized datasets generated and analysed during the current study are available from the corresponding author on reasonable request.
