# Supplementary material for: Metastases to the pituitary gland: insights from the German pituitary tumor registry
Source: Pituitary. 2023 Oct 30;26(6):708–15. doi: 10.1007/s11102-023-01361-0 (PMC10665242; doi:10.1007/s11102-023-01361-0)
Supplement: Supplementary file 1 — Supplementary Material 1 [file 11102_2023_1361_MOESM1_ESM.docx]

**Supplementary Table 1***Metastases to the sella region with unknown primary tumor*

| **#** | **Diagnosis** | **Suspected primary tumor** |
| --- | --- | --- |
| 1 | Poorly differentiated carcinoma of the sella region | Metastasis or primary tumor. (Suspected Germ cell tumor) |
| 2 | Metastasis of small cell neuroendocrine carcinoma. | Metastasis or primary tumor. (Differential diagnosis: pituitary carcinoma) |
| 3 | Immature carcinoma of the pituitary gland, not further classifiable | No reliable data on primary tumor |
| 4 | Poorly differentiated leiomyosarcoma | Metastasis or primary tumor |
| 5 | Highly differentiated adenocarcinoma | No reliable data on primary tumor |
| 6 | Sellar bone metastasis of poorly differentiated solid carcinoma. | Suspected bronchial carcinoma metastasis |
| 7 | Para- and intrahypophyseal metastasis of poorly differentiated solid carcinoma. | Suspected metastasis of small cell lung cancer |
| 8 | Poorly differentiated squamous cell carcinoma of the nasal mucosa | Metastasis or primary tumor. |
| 9 | Poorly differentiated neuroendocrine carcinoma. | Suspected bronchial carcinoma metastasis |
| 10 | Metastasis of a poorly differentiated carcinoma in a probably preexisting low-granular pituitary adenoma | No reliable data on primary tumor |
| 11 | Highly malignant sarcomatoid tumor | Metastasis of a renal cell carcinoma or a Fibrosarcoma or anaplastic meningioma |
| 12 | Infiltrate of poorly differentiated squamous cell carcinoma in the sella and sphenoid sinus mucosa. | Metastasis or primary tumor. |
| 13 | Metastasis of a well-differentiated keratinizing squamous cell carcinoma | No reliable data on primary tumor |
| 14 | Well-differentiated adenocarcinoma | Unclear, history of breast carcinoma |
| 15 | Metastasis of a solid carcinoma | Suspected bronchial carcinoma metastasis |
| 16 | Neuroendocrine carcinoma | Suspected metastasis of small cell lung cancer |
| 17 | Metastasis of a neuroendocrine carcinoma | No reliable data on primary tumor |
| 18 | Most likely metastasis of a neuroendocrine tumor in the sellar region | Suspected neuroendocrine tumor, NET G2 |
| 19 | Metastasis of moderately differentiated adenocarcinoma in the pituitary gland with inclusion of single, scattered STH-positive cells | No reliable data on primary tumor, likely metastasis to somatotropic adenoma. |
| 20 | Neuroendocrine tumor on the pituitary stalk | Metastasis or primary tumor.  (Differential diagnosis: Pituitary carcinoma) |
| 21 | Infiltrates or metastases from a poorly differentiated squamous cell carcinoma. | Suspected bronchial carcinoma metastasis |
| 22 | Non-adenomatous atypical tumor tissue and gonadotropic adenoma. | No reliable data on primary tumor, likely metastasis to adenoma. |
